# Homograft banking in Singapore: two years of cardiovascular tissue banking in Southeast Asia

**DOI:** 10.1007/s10561-012-9310-8

**Published:** 2012-04-27

**Authors:** Wee Ling Heng, Tracy Seck, Chiew Peng Tay, Alvin Chua, Colin Song, Chong Hee Lim, Yeong Phang Lim

**Affiliations:** 1 National Cardiovascular Homograft Bank, Department of Cardiothoracic Surgery, National Heart Centre Singapore, Singapore, Singapore; 2 Skin Bank Unit, Department of Plastic, Reconstructive & Aesthetic Surgery, Singapore General Hospital, Singapore, Singapore

**Keywords:** Heart valve transplantation, Homograft banking, Tissue banking, Antibiotic decontamination, Cryopreservation, Quality assurance

## Abstract

Established in 2008, the National Cardiovascular Homograft Bank (NCHB) has been instrumental in creating an available supply of cardiovascular tissues for implantation in Singapore. This article introduces its collaboration with Singapore General Hospital Skin Bank Unit. The procedure of homograft recovery, processing, cryopreservation and quality assurance are presented. Since its establishment, the NCHB has followed the guidelines set by the Ministry of Health Singapore and the American Association of Tissue Banks. A total of 57 homografts had been recovered and 40 homografts were determined to be suitable for clinical use. The most significant reasons for non-clinical use are positive microbiological culture or unsuitable graft condition. Crucial findings prompted reviews and implementation of new procedures to improve the safety of homograft recipients. These include (1) a change in antibiotic decontamination regime from penicillin and streptomycin to amikacin and vancomycin after a review and (2) mandating histopathogical examination since the discovery of cardiac sarcoidosis in a previously undiagnosed donor. Further, the NCHB also routinely performs dengue virus screening, for donors suspected of dengue infection. Cultural factors which affect the donation rate are also briefly explored. By 2010, 31 homografts had been implanted into recipients with congenital or acquired heart valve conditions. More than half of these recipients were children. Post-operative outcomes had been encouraging, with no report of adverse events attributed to implanted homografts.

## Introduction

Human heart valves have been used in surgical procedures for the past 50 years. Gordon Murray from Toronto reported the use of the first fresh aortic valve in 1956 (Murray 1956). In 1962, Donald Ross (1962) and Brian Barret-Boyes (1964; Bruce C. Paton 1992) had independently performed the first homograft implantation for their patients in London and Auckland respectively. In Singapore, the first human heart valve was implanted by Donald Ross and Ong Kim Kiat in 1985 (personal communication—Dr Ong Kim Kiat).

The first human heart valves were disinfected using chemical agents such as formaldehyde, glutaldehyde, beta propriolactone, ethylene oxide or high dose of antibiotics. Subsequently, prosthetic heart valves were introduced and used in the 1960s before xenografts came into existence in the 1970s (Parker 2010). However, due to the poor clinical durability and cell viability of chemically-treated homografts, this had led to a wane in interest in the use of such homografts for implantation (Gall et al. 1995), until low-dose antibiotic decontamination followed by cryopreservation and storage of homografts in liquid nitrogen was introduced.

Antibiotic-disinfected human heart valves are the replacement of choice in right ventricular outflow tract reconstructions and complex left ventricular outflow tract reconstructions in children and adults. It is also the first choice for replacements in patients with small aortic roots or aortic root endocarditis or patients requiring aortoventriculoplasties. Finally, it is the preferred alternative to prosthetic and bioprosthetic valves for patients where long-term post-operative anticoagulation therapy is contraindicated (Leeming et al. 2005), children, women of child-bearing age and adults with physically demanding occupations. The advantages of human heart valves include excellent haemodynamic performance, low thromboembolic rates without anticoagulation, no mechanical damage to blood cells which may occur for prosthetic valves, low incidence of post-operative endocarditis and symptomatic when there is a gradual wear-out of the valve (which is unlike the prosthetic valve which can fail shut suddenly) (Parker 2010).

To meet the increasing demand for aortic and pulmonary valve homografts and to reduce the over-reliance on expensive overseas sources, Singapore’s only cardiovascular homograft bank was established. The NCHB is located in a separate processing laboratory within the Singapore General Hospital (SGH) Skin Bank Unit (SBU)’s facility, as similar technologies are utilised for the processing and storage of skin and cardiovascular tissues. Furthermore, the donation of skin and cardiovascular homograft are governed by Medical (Therapy, Education and Research) Act (MTERA). This co-location has led to a greater synergy and better manpower sharing between the two tissue banks and has brought about a further development of a more conducive tissue banking environment in Singapore.

In this review, we present our methods of donor selection, recovery, processing, storage and quality assurance of the cardiovascular homografts. In the initial startup, the NCHB sought advice from QHVB, a heart valve bank that was established in 1969 (personal communication—Lisa Sparks, QHVB), and American Association of Tissue Banks (AATB).

## Methods

### Criteria for donors

The NCHB’s potential donors are aged between 3 and 65 years old. They belong to either one of the following categories: (1) tissue pledgers, (2) consent from the next-of-kin for brain dead and cardiac death donors or (3) explanted hearts from heart transplant recipients, whose valves are tested to be in working condition.

### Recovery of heart valve block

When a potential donor is identified, the recovery team is activated and arrangement for a suitable recovery site occurs. To minimise the risk of microbial contamination to the tissues, cardiovascular tissue recovery shall be (1) the exclusive activity that takes place in the recovery site (either a hospital’s operating theatre (OT) or a clean designated environment) at any one time and (2) completed within 15 h of cardiac death, if the donor is not refrigerated, and within 24 h, if refrigerated within 12 h (AATB, 12th edition).

Median sternotomy is performed with care not to enter the donor’s trachea or abdominal cavity which will contaminate the heart. A heart valve block, comprising of the aortic valve, pulmonary valve and the mitral valve leaflet is excised from the heart. The tissues are maintained in 1–10 °C saline for the entire dissection and transportation to the NCHB laboratory for maintenance of cell viability.

### Dissection, trimming and measurement of heart valves

Once the heart valve block reaches the laboratory, it is further separated into the aortic and pulmonary valves. This takes place under sterile conditions in the International Organisation for Standardisation (ISO) Class 5 certified laminar flow hood in an ISO Class 7 clean room. The tissue is kept moist in cold saline at temperature 1–10 °C to ensure the valve leaflets do not dehydrate during the entire dissection (Gall et al. 1995).

The valve annulus and length of conduit are measured. Coronary arteries are tied and ligated. The quality of homograft is evaluated prior to antibiotic decontamination. After recovery, competency of both the aortic and pulmonary valves is performed by flushing saline, with the help of a syringe, through the valves. The valve is deemed competent when there is neither leakage through the valves nor regurgitation as valve leaflets inflate.

For evaluation of valve quality, visual inspection for the presence of trauma, calcification, fenestration in the valves and the presence of atheroma and calcified deposits in the aortic wall is performed. Finally, the valve is evaluated and classified into one of the three categories (1) homografts with no visible abnormalities, (2) homografts with some imperfections but still acceptable or (3) non-clinical homografts (AATB, 12th edition).

### Antibiotic decontamination

From 2008 to 2009, recovered heart valves were decontaminated with the antibiotic cocktail of penicillin and streptomycin dissolved in Medium 199 (M199). Both aortic and the pulmonary valves were separately incubated at 37 °C for 6–12 h.

Since 2010, homografts are incubated in 100 μg/ml amikacin and 50 μg/ml vancomycin dissolved in M199, at 4 °C for 24–28 h.

### Cryopreservation

After antibiotic decontamination, the homografts are packaged under sterile conditions in the ISO Class 5 certified laminar flow hood in an ISO Class 7 clean room.

The homograft is then immersed in the freeze solution comprising of 10 % dimethyl sulphoxide (DMSO) in M199. DMSO penetrates the cellular membrane and prevents intracellular ice crystal formation that damages the tissue. Finally, the homograft and the freeze solution is packaged in double layers of transparent cryogenic bag and sealed together with the completed label insert. The packaged homograft is subsequently refrigerated, as DMSO is toxic to cells above 10 °C.

To monitor temperature changes that occur in the processed homograft, controlled rate freezing is performed using a controlled rate freezer in liquid nitrogen vapour, according to an electronically monitored profile. The rate of cooling is approximately −1 °C per minute. The homograft is then transferred to a quarantine liquid nitrogen storage tank when the temperature probe of the controlled rate freezing reaches −50 °C.

### Infectious diseases and microbiological testing

A cardiovascular donor is deemed unsuitable and the homografts discarded if any of the results for transmissible diseases, with the exception of venereal disease research laboratory (VDRL) test, is positive. A confirmatory treponema pallidum haemagglutination test is performed if the VDRL is reactive. The test for core total antibodies to hepatitis B antigen (anti-HBc total) is also mandatory. An additional test for hepatitis B surface antibodies (anti-HBs) is performed if the anti-HBc total turns out to be positive. The acceptable anti-HBs level is 10 miu/ml or above.

Screening for Human T-Lymphotrophic virus (HTLV), a retrovirus that is associated with T-cell lymphoid malignancies in adults, used to be mandatory for all cardiovascular homograft donors from 2008 to 2009. However, since 2010, mandatory serology screening for HTLV-I and II antibodies (anti-HTLV-I and II) is no longer performed. In addition, the following tests are also performed for donors who are suspected to have been infected, due to their prevalence in Southeast Asia: (1) Dengue flavivirus test using the real-time polymerase chain reaction method and (2) Mycobacterium tuberculosis test using acid-fast bacillus smear.

Microbiological cultures detect contamination by aerobes, anaerobes and fungus on the tissue and solution specimens post-recovery before antibiotic incubation and post-incubation prior to cryopreservation. This ensures that the NCHB releases only culture-negative homografts to the recipients.

### Histopathological examination

At the time of recovery of the cardiovascular homografts, samples of the atrial and ventricular myocardium, aorta and pulmonary artery are taken for routine histological examination. The tissue is routinely fixed and preserved in 10 % buffered formalin and wax embedment. Histological sections are at 5 microns and routinely stained with hematoxylin and eosin, Masson’s Trichrome for connective tissue and van Gieson technique for elastic tissue. The sections are examined by a trained pathologist for the presence of pathological conditions such as cystic medial degeneration or laminar necrosis of the aorta, cardiac muscle ischaemia and cardiomyopathy, presence and degree of inflammation and other clinically significant pathological feature.

### Quality assurance

For the safety of recipients, cryopreserved homografts are placed under quarantine until all the infectious diseases, microbiological and histopathological results confirm their clinical suitability. The NCHB’s quality assurance staff checks and verifies the results and donor records before submitting them for final review by the Medical Director.

After review, clinically suitable homografts are transferred to a clinical liquid nitrogen storage tank and made available for implantation. Cryopreserved homografts can be kept in liquid nitrogen for 5 years. Homografts that are clinically unsuitable are either stored in the quarantine liquid nitrogen storage tank for future research or discarded should there be non-consent for usage in research purpose.

### Thawing procedure

Homograft selected by the implant surgeon is transported to the OT of the implant hospital in a charged dry shipper at temperature below −135 °C.

In the OT, the homograft is thawed in saline that is warmed to approximately 37–42 °C. After it is completely thawed, a circulating nurse cuts the outer freezing bag. The scrub nurse then removes the sterile inner freezing bag containing the homograft. Subsequently, the homograft is rinsed in Lactated Ringers solution for 15 min to wash off the antibiotics and DMSO residues. This is a measure to reduce the potential of an allergic reaction due to antibiotics and DMSO residues when the homograft is implanted into a patient (Leeming et al. 2005).

Post-thaw tissue and solution microbiological cultures are the final step of quality assurance to verify that the homograft implanted into the recipient remains free from contamination. If the post-thaw result is positive, the homograft implant surgeon is notified immediately so that a prompt follow-up on his patient can be performed.

### Follow-up on homograft recipients

Regular follow-ups on the recipients are performed at 3 days, 3 months, 6 months, 1, 2, 3, 4 years and finally, 5 years post-implantation of the homografts.

The following information is obtained—the recipient’s current state of health, adverse reactions caused by homograft implant, the details of homograft explant, complications post-implant, further surgeries performed or scheduled and the previous and next check-up dates with the implant hospitals.

## Results

Between 2008 and 2010, the NCHB recovered 57 cardiovascular homografts (aortic, pulmonary, mitral and tricuspid valves, ascending and descending aorta and pulmonary patches) from 28 donors (Table 1). 40 homografts (70.2 %) were ultimately determined to be suitable for clinical use. The most significant reasons that caused unsuitability of homografts for clinical use were due to positive microbiological culture or unsuitable condition of the homografts such as regurgitation (Table 2).

**Table 1:** Type of donors from 2008 to 2010

Cause of death	Total no. of donors	% of donors
Brain death—multi-organ donors	10	35.7
Cardiac death	8	28.6
Live donor—explanted heart	10	35.7
Total	28	(100)

**Table 2:** Reasons for unsuitablity of homografts for implantation from 2008 to 2010

Reasons for unsuitability	Total no. of homografts	% of homografts
Abnormal histopathological findings	2	11.8
Technical error during separation of heart valve block	1	5.9
Unsuitable condition of homograft such as regurgitation	5	29.4
Positive serology	4	23.5
Failed microbiological culture	5	29.4
Total	17	(100)

Among the 36 cardiovascular homografts that were decontaminated with penicillin and streptomycin, 5 homografts (13.9 %) were initially tested positive for microbiological culture post-recovery. Eventually, in post-incubation culture, 2 were tested positive for bacterial culture and 1 was positive for fungal culture. In addition, a homograft was tested to be positive for MRSA in the post-recovery tissue culture but it was negative in the post-incubation culture. However, as penicillin and streptomycin are ineffective agents against MRSA, the homograft was discarded for the safety of recipients. This prompted a review in the effectiveness of penicillin and streptomycin against our local microflora. The SGH’s infectious diseases physicians were consulted and the use of amikacin and vancomycin for decontamination was subsequently proposed. The new antibiotic regime was implemented in 2010 after extensive validation studies were performed. Besides MRSA, the presence of *Clostridium*, *Streptococcus pyogenes*, other multi-resistant bacteria and fungus at any stage will also warrant the discard of a homograft. For the 21 homografts decontaminated with amikacin and vancomycin, 9 homografts (42.9 %) were tested positive for microbiological culture post-recovery and no homograft was tested positive for bacterial culture after antibiotic decontamination. A homograft was tested positive for fungal culture (Table 3).

**Table 3:** Type of micro-organisms isolated in the microbiological cultures of the cardiovascular homografts from 2008 to 2010

Type of micro-organisms	Post-recovery tissue	Post-recovery solution	Post-incubation tissue
*Propionibacterium acnes*		6	1
*Pseudomonas* species	1		
Coagulase-negative *Staphylococcus*	1	3	
Coagulase-positive *Staphylococcus*	2	1	
*Escherichia coli*	1		
*Acinetobacter* species	1		
*Micrococcus* species			1
*Staphylococcus aureus*		2	
^b^MRSA	1		
*Rhodococcus* species	1		
*Alpha Haemolytic Streptococcus*	1		
*Bacillus* species	1		
^a^ *Candida albicans*			1
^a^ *Candida parapsilosis*			1

^a^Refers to fungus

^b^Refers to bacteria, which when isolated at any stages of processing, will have to be discarded. These micro-organisms were MRSA, *Clostridium* species, *S. pyogenes* and multi-resistant bacteria

A significant observation is there was a higher incidence of positive microbiological tissue and solution culture post-recovery for homografts recovered from cardiac death donors (50 %), followed by brain dead multi-organ donors (37.5 %) and heart transplant recipients (12.5 %).

Of the 57 cardiovascular homografts that were determined to be suitable for clinical use, 31 were released for implant to 30 recipients from different restructured and private hospitals in Singapore. Most of the homografts released were aortic and pulmonary valves, which were in extremely high demand amongst cardiac surgeons. The low number of homograft release is primarily limited by a shortage of these two types of valves.

A local and foreign waiting list was also implemented in July 2009. This waiting list facilitates a more systematic release of homografts, ensuring first-come-first-release of available homografts and a priority in allocation for Singaporeans. However, in rare cases of clinical urgency, the NCHB Medical Director will decide on the release of reserved homografts. There has been a rise in the number of recipients waiting for a homograft implant on the waiting list. This could be attributed to an increase in awareness among cardiac surgeons that a local source of homografts is available.

Among the recipients who received the NCHB’s homografts, 53.3 % were paediatric recipients below the age of 18 years and 40 % were adults who suffered from congenital heart diseases. The remaining 6.7 % were adults who underwent operation due to infective endocarditis (Table 4). Till date, follow-up of 30 recipients have been performed.

**Table 4:** Clinical diagnosis of the NCHB recipients from 2008 to 2010

Diagnosis of recipients	No. of recipients
Congenital	
Tetralogy of fallot	6
Congenitally corrected transposition of the great arteries	1
Transposition of the great arteries	1
Pulmonary atresia with ventricular septal defect	6
Absence of pulmonary valve syndrome	1
Pulmonary regurgitation	5
Stenosis	7
Acquired	
Infective endocarditis	2
Total	30

## Discussion

Since the start of the NCHB’s operation, it has been facing a chronic shortage of tissues. This is due to several reasons.

Firstly, the invasiveness of recovery surgery and deep-rooted cultural beliefs result in many non-consent from next-of-kin of deceased donors. The Asian religious and cultural beliefs in afterlife contribute to many non-consent, as relatives do not want the deceased to be without an intact body in the netherworld (Chua et al. 2007). Moreover, it is not in the Asian culture to discuss about the topic of death with their family members, as it is regarded as inauspicious. As a result, it is not uncommon for the relatives to be unaware of the decision of whether their deceased loved ones wish to donate their organs/tissues upon death. Relatives’ uncertainties, together with the additional time frame required for the recovery of homografts, the next-of-kin would usually not consent for donation of their deceased loved ones’ tissues.

In Singapore, the donation of cardiovascular homograft is covered by MTERA. Unlike Human Organ Transplant Act (HOTA) which mandates the donation of heart, liver, kidney and corneas for all Singaporeans and Singapore Permanent Residents (PR) age 21 years and above unless one opts-out from the act, MTERA is the voluntary donation of other organs/tissues for anyone regardless of nationality and who is 18 years old and above. There are two ways a MTERA opt-in consent are obtained: (1) an individual, aged 18 years and above, signs a pledge form or (2) consent from next-of-kin of a donor who had not pledged his organs/tissues under MTERA before passing away. Currently, consent obtained through next-of-kin of donors is significantly higher than from tissue pledgers. Surveys performed by the NCHB, during public awareness events, revealed that most members of public were unaware of the existence of MTERA and had a misconception that heart valve donation was covered under HOTA. As a result, many people did not actively make a decision to pledge the donation of their cardiovascular tissues. Therefore, to increase the overall donation rate in Singapore in the long term, consistent efforts in public education, especially in reaching out to the young generation and dispelling a common misconception by reinforcing that heart valve donation is under MTERA and not HOTA, is necessary. However, this will take a number of years, probably even a generation or more, before any increase in consent rate or number of pledgers can be observed.

Secondly, some homografts were discarded as a result of contamination during recovery. A significant observation for the highest incidence of positive microbiological tissue and solution culture post-recovery for homografts from cardiac death donors are largely due to a difference in recovery sites. Data revealed that recovery performed in non-OT clean environment for cardiac death donors, resulted in a higher post-recovery contamination rate of 62.5 %. In contrast, both the recovery for brain-death multi-organ donors and the heart transplant patients are performed in the OTs. Nonetheless, despite tissue recovery being performed in the OTs, the incidence of contamination post-recovery remains significantly higher for homografts recovered from multi-organ donors (37.5 %), as compared to heart transplant patients (12.5 %). This lower post-recovery contamination rate in homografts recovered from heart transplant patients can be explained by several reasons: (1) a shorter exposure time to the OT environment, (2) the complete isolation of thoracic cavity from abdominal cavity during heart transplantation (Gall et al. 1995; Tabaku et al. 2004), (3) an absence of the inherent problem of contamination caused by microbial migration from the gastrointestinal tract through the bloodstream to the heart valves after death (Gall et al. 1995) and (4) a shorter hospitalization stay prior to tissue recovery.

Thirdly, some homografts are tested to be unsuitable after a stringent screening process. According to the AATB’s recommendation, after 11 November 2009, screening for anti-HTLV-I & II was no longer mandatory for all cardiovascular homograft donors, as it is a non-leukocyte-rich tissue. Because of this, testing shall be determined by the Medical Director (AATB, 12th edition). Hence, since 2010, the NCHB ceased its mandatory screening. Furthermore, it is noteworthy that the incidence of HTLV infection follows a unique geographical and ethnic distribution (Wang et al. 1991), in which Singapore does not fall within the HTLV-endemic region (Zhao et al. 1991). In addition, screening for anti-HTLV-I and II was not performed for solid organ donors in Singapore.

The NCHB performs two tests which are not commonly performed in other tissue banks. The first test is for detection of dengue virus, which is transmitted by flavivirus-infected Aedes mosquitoes. It is a mandatory test for all donors who manifest symptoms of dengue infection. This is because dengue infection, which causes dengue fever, dengue haemorrhagic fever or dengue shock syndrome, is endemic in many parts of Southeast Asia, including Singapore. The high prevalence in dengue seropositivity among the adult population in Singapore was revealed through a study conducted by Wilder-Smith et al. that 45 % of the adults had positive dengue serology (Wilder-Smith et al. 2004).

In solid organ transplantation, the transmission of dengue infection from donor to recipient can result in a loss of the allograft and even death of the recipient in severe cases. The occurrence of dengue virus infection in an immunocompromised transplant recipient can have detrimental effects, the most life-threatening of these being the development of dengue shock syndrome. (Tan et al. 2005). It was first reported in 2004 that two renal transplant patients passed away after each of them received a kidney from a donor who was infected by the dengue virus. Both patients eventually developed complications and died of multi-organ failure within weeks after their transplantation. (Ng and Chan 2010). Therefore learning from this experience, the NCHB takes precaution by screening all cardiovascular homograft donors who manifest any one of the signs of suspected dengue infection: (1) prolonged prothrombin time or partial thromboplastin time which indicates an abnormality in the coagulation pathways, (2) low platelet count of below 140 × 10^9^ or (3) platelet count falls below 25 % of their own normal baseline, which is between the range of 140 × 10^9^–440 × 10^9^.

The second test is histopathological examination of the donor heart. Before December 2008, mandatory histopathological examination was not performed on recovered tissues. Only the results of histopathological examination on explanted hearts, which was the hospital’s routine test for heart recipients, were evaluated. Subsequently, this procedure was reviewed when one of the explanted hearts was diagnosed with granulomatous myocarditis, with features suggestive of cardiac sarcoidosis. Cardiac sarcoidosis is a rare but potentially fatal condition of unknown etiology that has been reported to occur in isolation without evidence of sarcoidosis in other parts of the body. Sarcoid granuloma may involve any location in the heart, including the myocardium, endocardium and valve leaflets leading to regurgitation (Kim et al. 2009). The principal sequelae includes conduction abnormalities, arrhythmias, heart failure and even sudden death (Ayyala et al. 2008). A study in Japan revealed the mortality related to cardiac sarcoidosis was discovered in 46.9 % of sarcoidosis-related deaths. However, clinical diagnosis had only been made in 26.7 % of the cases (Iwai et al. 1993), revealing the disease’s variable clinical manifestation (Kim et al. 2009). Therefore, since 2009, mandatory histopathological examination is implemented in the NCHB. Further, we also recommend all cardiovascular tissue banks to consider performing this examination. The benefits for histopathogical examination of the heart tissues include the identification of any potentially inherited or undetected heart condition that will compromise valve quality and eventually, on the recipient’s health.

The ultimate objective of a reliable cardiovascular bank should not solely focus on increasing the number of donors and cardiovascular tissues recovered. More importantly, there must be a stringent quality assurance programme in place, which should be constantly reviewed, to ensure that the homografts recovered, processed and stored, are safe for the recipients who require them.
